# Ultrasonography of sudden swollen tongue in a calf

**DOI:** 10.1186/s12917-020-02398-0

**Published:** 2020-06-16

**Authors:** Takeshi Tsuka, Yoshiharu Okamoto, Yuji Sunden, Takehito Morita, Masamichi Yamashita, Tomohiro Osaki, Kazuo Azuma, Takao Amaha, Norihiko Ito, Yusuke Murahata, Tomohiro Imagawa

**Affiliations:** Department of Veterinary Clinical Medicine, School of Veterinary Medicine, Faculty of Agriculture, Tottori University, 4–101, Koyama-Minami, Tottori, Japan

**Keywords:** Cattle, Glossectomy, Snake bite, Tongue, Ultrasonography

## Abstract

**Background:**

In cattle, the lingual diseases are primarily diagnosed postmortem by histopathological examination of the affected tongues obtained after the death or during necropsy. In humans, ultrasonography has been used to provide differential diagnoses, and for preoperative or intraoperative planning of glossectomy in various lingual diseases. This is a bovine clinical case report, in which ultrasonography for sudden swelling of the tongue, which was possibly caused by snake bite, was utilized as a preoperative indication to perform a glossectomy.

**Case presentation:**

An eight-month-old female Japanese black calf presented with sudden swelling of the tongue with well-defined discoloration in the cranial region. A 10-MHz linear probe on a portable-type ultrasound machine (MyLabOne VET, Esaote Co., Genova, Italy) was applied to the ventral surface of the tongues in the affected case, and also in five healthy calves under sedation to observe normal tongues. Ultrasonography of the swollen tongue in this case revealed that the ventral lingual muscular layers were severely thickened compared with those of normal tongues. However, the muscle layers were regularly aligned with the echogenic muscular fibers. This resembled the lingual muscular architectures of normal tongues. Color-flow Doppler ultrasonography revealed that blood flow was weakened in the small peripheral vessels in the spaces between the lingual muscular structures, and was lacking in the deep lingual artery between the apex and base of the tongue. This finding was very different than that of normal tongues, which exhibited weakened or rich blood flows. Based on ultrasonographic findings, this case was treated with glossectomy. After recovery, the calf grew up normally with a normal appetite and rumination, and did not exhibit mouth pain behavior. Histopathologically, hemorrhagic necrotic changes, together with focal formation of fibrin thrombus in the lingual blood vessels in the affected tongue, were noted.

**Conclusions:**

To the best of our knowledge, the present report is the first description of lingual ultrasonography performed in cattle. In this case, ultrasonography enabled visualization of decreased vascularity, which might be associated with hemorrhage or formation of fibrin thrombus in the suddenly swollen tongue presented.

## Background

Severe and sudden swelling of the tongue is rarely observed in cattle. The most common cause is infection by *Actinobacillus lignieresii*, resulting in development of a granulomatous swollen tongue known as “wooden tongue” caused by actinomycosis [1]. The other primary cause is various types of tumors arising from the tongue, such as ameloblastic fibroodontoma, mast cell tumor, melanoma, rhabdomyosarcoma, and squamous cell carcinoma [2]. Snake bite is another possible cause of lingual swelling, based on a previously reported case in a camel [3], although this has not previously been reported in cattle. The cause of tongue swelling in cattle has historically been diagnosed by clinical symptoms, various laboratory tests, and most commonly by histopathological examination after death or during necropsy of the animal [1, 2]. To our knowledge, ultrasonography has never been used as an antemortem diagnostic method in bovine practice, despite previous postmortem use for bovine tongues and clinical uses for other animals [4, 5].

Ultrasonography enables the visualization of the shape, position, and movements of the tongue [6]. In addition, Doppler ultrasonography has been utilized to evaluate the vascularity of lingual cancers and pyogenic granuloma [7–9]. The purpose of this case report was (1) to compare the affected tongue with normal tongues based on the blood flow and visual state of the structures, (2) to present the diagnostic process using ultrasonography and the subsequent performance of glossectomy on this bovine case of sudden swelling of the tongue, and (3) to discuss the role of ultrasonography in diagnosis of lingual diseases of cattle based on its prior application in human case reports.

## Materials and methods

### Ultrasonographic imaging procedure for the bovine tongue

Ultrasonographic examinations were carried out using a portable ultrasound machine (MyLabOne VET, Esaote Co., Genova, Italy). A linear probe was used with an ultrasound frequency range of 10–13 MHz. The measurement accuracy of the probes was 0.1 mm. Bovine tongues were examined the following ultrasound conditions: scan depth 40 mm, dynamic range 79 dB, and the highest level of time gain compensation (Gain 100%). The probe was applied longitudinally (apex to base) on the ventral surface of the tongue, which was pulled out manually from the mouth under sedation with intravenous injection of xylazine hydrochloride (0.2 mg/kg) in five healthy calves and the affected calf with a swollen tongue. In addition, the probe was applied on the dorsal surface of the tongue of the affected calf in standing position (without sedation).

Ventral muscular thickness (VMT), distance between the ventral surface of the tongue and the deep lingual artery (DSA), and the width of the colored region on the Doppler image of the deep lingual artery (WD) were measured in the middle and ventral regions of the tongue.

### Healthy calves

Five Japanese black calves bred at the Tottori University farm and aged between six and 8 months were used as healthy controls. Examinations were performed after sedation in the recumbent position. Soon after the 5–10 min procedures, intravenous injection of atipamezole hydrochloride (an α2 adrenergic receptor antagonist; 0.02 mg/kg) was used to reverse the sedation, allowing rapid waking in all five animals followed by normal behavior, including eating hay and concentrates without any abnormal movement of the tongue.

### Affected calf

**Fig. 1:**
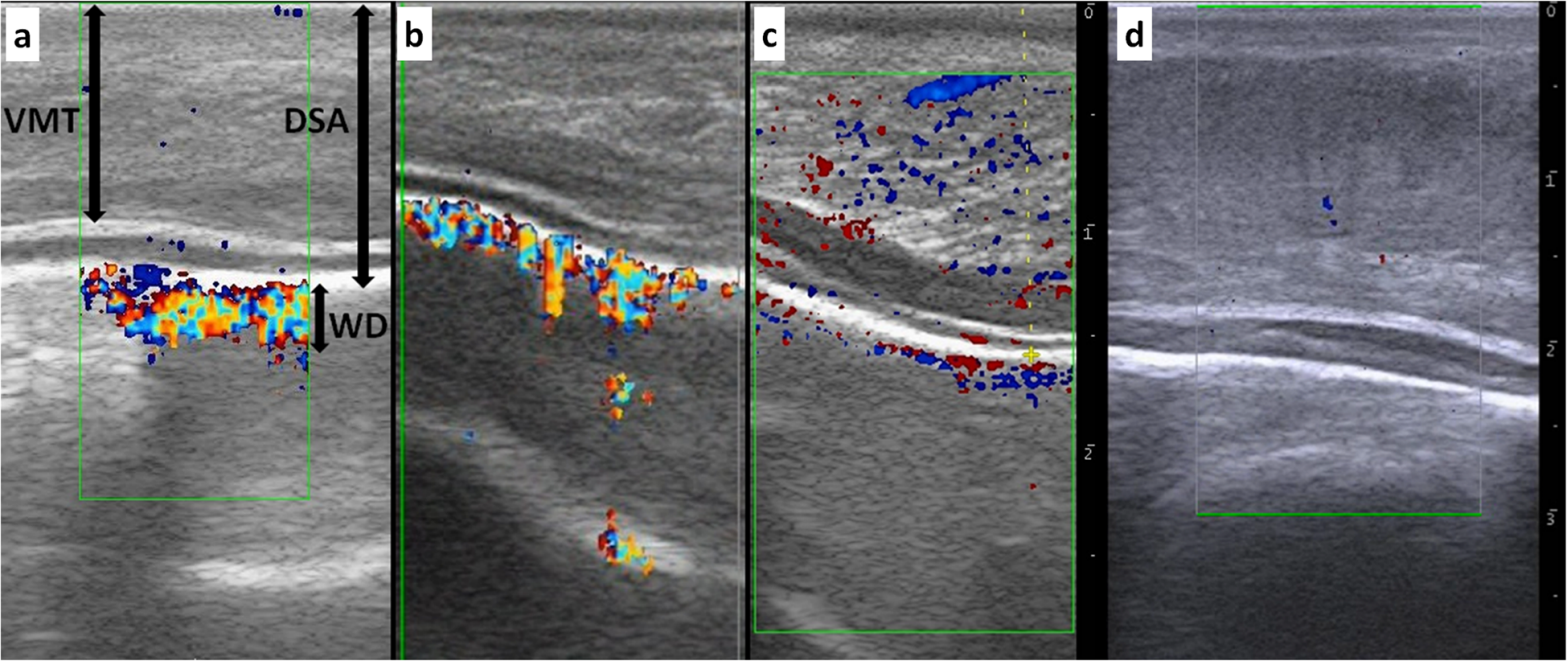
Sagittal ultrasonographic images of the tongue ventral surfaces in three of five healthy calves (**a-c**), and the affected case (**d**). **a,b** Normal ventral lingual structures exhibit 1) muscular layers in which the echogenic muscular fibers are regularly aligned, 2) two hyperechoic lines, which include homogenous hypoechoic structure, and 3) the deep lingual artery running along the distal hyperechoic line. Lingual structures deeper than the hyperechoic lines disappear due to a reverberation artifact. Rich blood flow is noted in the deep lingual artery on the Color-flow Doppler ultrasonograms. VMT: ventral muscular thickness. DSA: distance between the ventral surface of the tongue and the deep lingual artery. WD: width of the colored region on Doppler image of the deep lingual artery. **c** Blood flow is weakened into the deep lingual artery, but is comparatively rich in the intramuscular vessels on the Color-flow Doppler ultrasonogram. **d** The ventral lingual muscular layers are thicker than that of the normal tongue, but retains the regular alignment of the echogenic muscular fibers. Blood flow is not evident into the deep lingual artery on the Color-flow Doppler ultrasonogram. Scale: 10 mm

An eight-month-old female Japanese black calf presented with sudden swelling of the tongue for 5 days prior to admission. Two small holes were present in the apex and ventral surface of the tongue on immediate examination, but had disappeared by admission. Discoloration of the lingual mucosa was noted along the entire circumference of the cranial half of the tongue (Fig. 1a). The discoloration exhibited clear and oblique border, separating it from the normal lingual mucosa on the caudal half of the tongue, and extended towards the base at the right edge of the tongue. The discolored lingual mucosa was dry, and was easily peeled off manually. The softness in the discolored region was the same as the normal mucosa. The tongue was swollen in the dorsal-ventral direction, and could not be normally put into the mouth. The cranial half of the tongue drooped outside of the mouth. The calf could not use the affected tongue for eating food or drinking, despite a strong appetite. Continuous administration of antibiotics did not reduce the lingual swelling or improve clinical signs.

Hematological examination revealed moderately elevated white blood cell count (15,000/μl; reference value: 6500-11,500/μl), and normal platelet count (79.5 × 10^4^/μl: reference value 68.1 ± 25.2 × 10^4^/μl) [10]. Highly elevated creatine kinase (1629 U/l; reference value: 29.8–302.5 U/l) was evident on serum biochemical examination [10].

## Results

### Ultrasonography of healthy bovine tongues

**Fig. 2:**
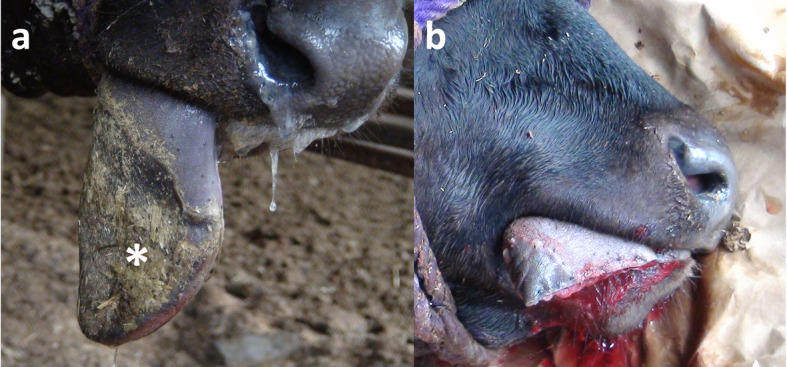
Gross appearance of the tongue at 5 days after onset of swelling (**a**), and soon after surgery (**b**). **a** The severely swollen tongue is discolored in the cranial half (asterisk). **b** The tongue is shortened due to removal of the discolored cranial region

The bovine tongue architecture could be visualized more clearly using as ultrasound frequency of 10 MHz compared with the higher ultrasound frequencies. The ventral muscular layers exhibited multilayered echogenic lines running along the ventral surface or obliquely (Fig. 1a-c). Small vessels were present periodically within the spaces between the echogenic muscular fibers. Three hyperechoic lines ran between the apex and base along the deeper edge of the ventral muscular layers. Of the three hyperechoic lines, the deeper linear structure appeared to be thicker and more hyperechoic when compared with the closer two echogenic lines. The spaces between the three lines were comparatively hypoechoic relative to the ventral muscular layers. The deep lingual artery ran along the deeper hyperechoic line. Color-flow Doppler ultrasonography revealed that blood flow was rich into the deep lingual artery in four of the five normal tongues (Fig. 2a,b), but was weakened in one of the five tongues (Fig. 2c). Lingual structures deeper than the hyperechoic lines disappeared due to a reverberation artifact. The average TSM was 12.5 mm (range, 10.0–15.0 mm). The average DSA was 14.6 mm (range, 11.6–16.9 mm). The average WD was 4.4 mm (range, 1.2–7.1 mm).

### Ultrasonography of the swollen tongue

When the probe was applied to the dorsal surface of the swollen tongue, the dorsal muscular layers ran uniformly and obliquely from the dorsal-base to the ventral-apex direction along the discolored mucosa on the cranial half of the tongue, and resembled the muscular layers present along the normal mucosa on the caudal half of the tongue. However, echogenicity in the cranial half of the tongue was clearly decreased compared to the caudal half of the tongue. Examination of the base and ventral surfaces of the tongue was difficult without sedation, as the calf moved and retracted the tongue during examination. When the probe was applied to the ventral surface of the tongue, there was no obvious destruction to the ventral muscular layers. Multilayered echogenic lines were observed running obliquely, and were identical to the normal lingual structures (Fig. 2d). The VMT was 17.8 mm, indicating that the ventral muscular layers in the tongue of the affected calf were thicker compared to normal bovine tongues. Color-flow Doppler ultrasonography revealed that blood flow was present in parts of the small peripheral vessels in the spaces between the oblique-running lingual muscular structures. However, lack of blood flow was detected within the deep lingual artery between the apex and base of the tongue. The DSA was 22.0 mm. The WD could not be measured, as the colored blood flow region was not evident on the Color-flow Doppler ultrasonogram of the artery.

### Treatment and clinical outcomes in the affected calf

Ultrasonographic findings indicated that lack of blood flow might contribute to the discoloration observed on the cranial half of the tongue. Therefore, a glossectomy was performed according to a previously reported surgical procedure [11]. Briefly, a tourniquet made of nylon string was applied proximal to that cranial half of the tongue for which resection was recommended based on gross and ultrasonographic examinations. The tongue was transected in the proximal region rather than on the border between the discolored and normal lingual mucosa. However, a portion of the discolored lingual structure in the right edge of the tongue could not be removed, because it extended towards the base. A slight hemorrhage was present on cut surface. The transected cut surface was trimmed to a V-shape such that the dorsal and ventral aspects were protruding, rather than the center portion of the tongue. The ventral and dorsal cut surfaces were fixed together to cover the center with interrupted horizontal mattress suturing using an absorbable suture material (MAXON 3–0, Davis & Geck, USA). Final closure of the ventral and dorsal surfaces was achieved by intradermic suturing with an absorbable suture material (Fig. 1b).

Immediately after surgery, the animal could put the tongue back into the mouth, and close the mouth. The animal was treated with seven-day intravenous administration of antibiotics postoperatively. The animal was offered hay on day three after surgery, followed by a gradual provision of concentrates. The animal could grip hay by rolling of the shortened tongue, and eat concentrates by burrowing the lip into the food. The animal developed a normal appetite and rumination without exhibiting mouth pain behavior; and grew up normally.

**Fig. 3:**
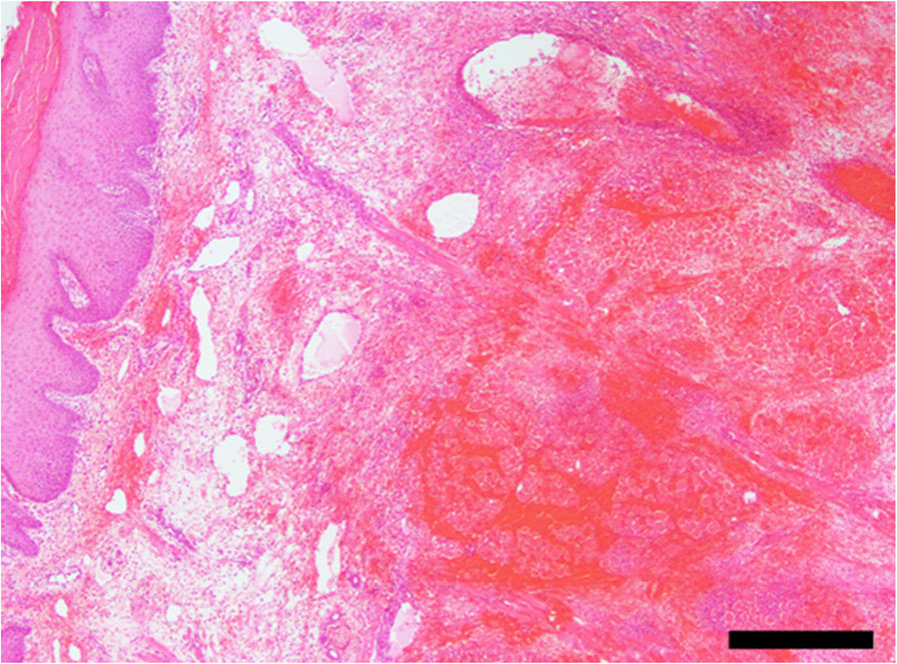
Histopathology of the swollen tongue The submucosal tissue is edematous and a marked necro-hemorrhagic lesion in the glossal skeletal muscles is noted (HE). Bar = 500 μm

**Fig. 4:**
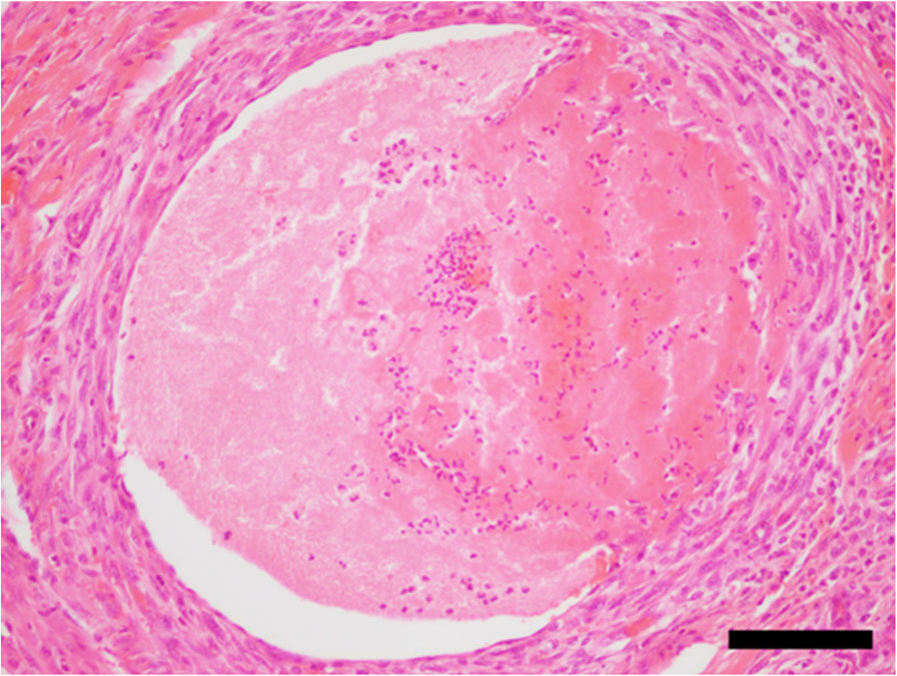
Histopathology of the swollen tongue. The thrombus is adherent to the vascular walls, and is accompanied by fibroblastic cells (HE). Bar = 1000 μm

The excised portion of the tongue was fixed in 10% neutral-buffered formalin and embedded in paraffin wax. Sections were cut at a thickness of 5 μm, and stained with hematoxylin and eosin. Extensive diffuse hemorrhage, degeneration, and necrosis were evident in the muscular fibrous tissues (Fig. 3). The necrotic muscular tissues appeared to have partly regenerated. Necrosis was also observed in parts of the mucosal epitheliums. The muscular fibrous tissues were extensively infiltrated with inflammatory cells such as macrophages, neutrophils, lymphocytes, and eosinophils. Calcification was present with the infiltration of inflammatory cells. Fibrin thrombus was present in the peripheral vessels within the muscular structures (Fig. 4). The thrombus adhered to the vascular walls, and was accompanied by fibroblastic cells. No area with the shape of an inflicting snake fang was detected histologically. These findings supported the diagnosis of extensive hemorrhagic necrosis of the tongue.

## Discussion and conclusions

Severe swelling of the tongue has rarely been observed in cattle, but is a typical clinical sign of various lingual diseases, such as actinomycosis and lingual tumors [1, 2]. Lingual snake envenomation was initially suspected in this case, as the lingual swelling was acute, and two small punctures were present on the ventral surface of the tongue at the time of injury. In addition, the clinical signs, such as the well-defined discoloration in the cranial half of the swollen tongue, resembled those of a previous case of a camel with lingual snake envenomation [3]. The diagnosis of snake bite is commonly obtained from the gross discovery of snake fang punctures or histopathological views of the shape of the inflicting fang [12]. However, this histopathological finding is difficult to detect in snake bite cases, as the lesions are very small relative to the size of the tongue [12]. The application of ultrasonography for this case clarified the lingual pathological changes due to suspected snake envenomation.

In humans, ultrasonography has been performed in the investigation of cases with abnormal speech, and for the diagnosis of lingual diseases [6–9, 13]. Application of ultrasonography in lingual diseases of cattle has not been reported, although previous postmortem use for slaughterhouse specimens as a model of human lingual diseases was reported [4]. Because this prior report did not describe the ultrasonographic appearance of normal bovine lingual structures [4], the present case study is supported by additional assessments, including a point of technical view and normal ultrasonographic appearance.

Our scanning technique, in which the probe was placed directly on the ventral or dorsal surface of the tongue, differs from other previously reported techniques, in which the probe is placed on the submental skin surface in humans and other animals [4, 5]. However, in cattle, the larger size of the mandible could interfere with visualization of the lingual structures by the submental approach, as evidenced by a prior finding that the submental technique did not allow visualization of the tongue apex in horses due to the strong acoustic shadows generated by the mandible [5]. When ultrasonography was applied to the standing, non-sedated calves, the lingual structures of the dorsal surface could be visualized, but the ventral surface and base of the tongue could not be visualized. Tongue movement precludes visualization of the ventral surface and base of the tongue, even in humans [13]. In addition, artifacts caused by motion of the tongue frequently prevent clear visualization of the lingual structures in humans [13]. The ventral surface of the bovine tongue could be recommended as the ideal diagnosable location, as applying the probe allows visualization of the deep lingual artery. The deep lingual artery runs closer to the ventral surface than the dorsal surface of the bovine tongue [14]. The deep lingual artery is bilaterally present within the anatomical space between the dorsal muscular layers (working as the intrinsic muscles) and ventral muscular layers (mainly consisting of Styloglossus muscle working as the extrinsic muscles) in the middle and base, and commonly terminates by an anastomosis of the right and left deep lingual arteries in the apex of the bovine tongue [14]. Therefore, the probe must be turned gently toward the left or right from the midline of the tongue for visualization of the bilateral deep lingual arteries. In addition, the blood flows in the deep lingual artery cannot be visualized on the Color-flow Doppler ultrasonograms without exactly placing the probe on the ventral surface of the tongue [13]. In one of the five healthy calves evaluated, Color-flow Doppler ultrasonogram might have detected weakened blood flow in the deep lingual artery due to handling instability and failure to place the probe in the exact scanning position toward the bilateral structures. Thus, sedation is recommended for ultrasonographic examination of the bovine tongue to allow efficient handling of the applied probe, improving visualization accuracy of the deep lingual artery via the ventral surface of the tongue.

The primary color-flow Doppler ultrasonographic characteristic of the affected tongue in this case was lack of blood flow in the deep lingual artery, which might have been caused by damage to the artery, resulting in extensive hemorrhage and local thrombosis of the lingual structures [15]. The decreased vascularity might have contributed to the well-defined discoloration in the cranial half of the tongue. The discoloration resembled findings of local lingual thrombosis and infarction following the progression of necrotic changes in previous human cases [16]. In addition, damage to both deep lingual arteries running parallel to the tongue is a possible cause of lingual necrosis in humans [15]. Unfortunately, it could not be concluded whether both deep lingual arteries were damaged, as it was not clear as to which of the two arteries was visualized on sagittal scanning, and both arteries could not be visualized on the same ultrasonogram.

Lingual snake envenomation, as the possible cause of this case, could have induced extensive overall damage to the blood vessels of the tongue. *Gloydius blomhoffii*, commonly known as mamushi, is a venomous pit viper species endemic to Japan [17]. The venom causes increased capillary permeability, resulting in internal hemorrhages and thromboembolisms associated with damage to the vasculature [12]. Unilateral or bilateral changes to the vascularity of the deep lingual artery due to angiogenesis and changes of vessel width with vessel compression is characteristic of lingual tumors [8, 15]. This leads to an imbalance of vascularity and vessel width between the two deep lingual arteries of the tongue [7]. This abnormality can be visualized when the ultrasound probe is applied transversely to the tongue [7]. Hence, when the bovine tongue requires examination with ultrasonography, the ultrasound probe must be applied both transversely and sagittally to the surface of the tongue.

Ultrasonography did not allow discernible visualization of the hemorrhagic necrosis of the lingual structures in this case, as the lingual muscles maintained regular layering of the echogenic structures on the ultrasonogram. When ultrasonography was previously applied in a bovine case with a swollen mandible due to snake envenomation, the extensive necrotic lesion exhibited irregular hyperechoic parenchymal structures, including a large heterogenic hypoechoic focus on day eight after injury [17]. The echogenicity of the necrotic structures could vary with time after injury or the healing process after any primary cause, including thrombosis and snake envenomation [18]. The five-day interval between swelling onset and examination in this case might have been too short for the development of extreme destruction in the lingual structures.

The most common cause of swollen tongue in bovines is infection with *Actinobacillus lignieresii*, resulting in the development of a granulomatous changes [1]. Lingual tumors lead to the formation of nodules within the lingual structures, or protrusions from the lingual membranes in cattle [2]. If ultrasonography is used for these cases, nodular and granulomatous structures would likely be indicated by a well-marginated mass in the muscular fibers aligned irregularly, as in previous human case reports [8, 9].

Glossectomy was performed for the present case, as the lingual lesion was presumed to be irreversible based on the ultrasonographic findings indicating lack of blood flow in the deep lingual artery, as well as the clinical history and gross severity. Surgical excision and refreshment are considered to be one of the most successful treatments for swollen tongues caused by glossoplegia, lingual trauma, and snake bite in large animals [3, 11, 12]. However, surgical excision might not be recommended for treatment of bovine actinomycosis-induced wooden tongue associated with granulomatous changes [1].

Ultrasonography is an imaging tool applicable to the preoperative differential diagnosis process for various lingual diseases, as it can visualize the internal structure and vascularity of the lesion [13]. For this application, lingual vascularity should be assessed by objective evaluation methods, such as pulsatility index and ultrasound velocity, instead of the subjective method performed in the previous and present reports [8, 9, 18].

## Data Availability

Not applicable.

## Abbreviations

DSA: The distance between the ventral surface of the tongue and the deep lingual artery
VMT: Ventral muscular thickness
WD: The width of the colored region on Doppler image of the deep lingual artery

## References

[1] Rycroft AN, Garside LH (2000). Actinobacillus species and their role in animal disease. Vet J.

[2] Gangwar AK, Devi KS, Kumar V (2016). Diagnosis and management of lingual Rhabdomyosarcoma in a bullock. Intas Polivet.

[3] Sadan MA (2017). Surgical treatment of some tongue affections in camels (*Camelus Dromedarius*). J Agr Vet Sci.

[4] Izzetti R, Fantoni G, Gelli F, Faggioni L, Vitali S, Gabriele M, Caramella D (2018). Feasibility of intraoral ultrasonography in the diagnosis of oral soft tissue lesions: a preclinical assessment on an ex vivo specimen. Radiol Med.

[5] Solano M, Penninck DG (1996). Ultrasonography of the canine, feline and equine tongue: Normal findings and case history reports. Vet Radiol Ultrasound.

[6] Gick B (2002). The use of ultrasound for linguistic phonetic fieldwork. J Int Phon Assoc.

[7] Kimura Y, Ariji Y, Gotoh M, Toyoda T, Kato M, Kawamata A, Fuwa N, Ariji E (2001). Doppler sonography of the deep lingual artery. Acta Radiol.

[8] Yamamoto C, Yuasa K, Okamura K, Shiraishi T, Miwa K (2016). Vascularity as assessed by Doppler intraoral ultrasound around the invasion front of tongue cancer is a predictor of pathological grade of malignancy and cervical lymph node metastasis. Dentomaxillofac Radiol.

[9] Cantisani V, Del Vecchio A, Fioravanti E, Romeo U, D'Ambrosio F (2014). Color-Doppler US features of a pyogenic granuloma of the upper dorsum tongue. J Ultrasound.

[10] Morita Y, Sugiyama S, Tsuka T, Okamoto Y, Morita T, Sunden Y, Takeuchi T (2019). Diagnostic efficacy of imaging and biopsy methods for peritoneal mesothelioma in a calf. BMC Vet Res.

[11] Ducharme N, Fubini SL, Ducharme N (2004). Surgery of the bovine digestive system. Farm animal surgery.

[12] Banga HS, Brar RS, Chavhan SG, Saudhu HS, Kammon AM (2009). Pathology of snake bite in cow. Toxicol Int.

[13] Sugawara C, Takahashi A, Kawano F, Kudo Y, Ishimaru N, Miyamoto Y (2016). Intraoral ultrasonography of tongue mass lesions. Dentomaxillofac Radiol.

[14] Ferreira J, Nogueira DJ, Rodrigues BF, Alvarenga BF (2009). Blood supply in the tongue of Nellore Bos indicus (Linnaeus, 1758). Anat Histol Embryol.

[15] Mun MJ, Lee CH, Lee BJ, Lee JC, Jang JY, Jung SH, Wang SG (2016). Histopathologic evaluations of the lingual artery in healthy tongue of adult cadaver. Clin Exp Otorhinolaryngol.

[16] Fernandez-Casado A, Sanchez-Gonzalez B (2009). Images in clinical medicine. Tongue necrosis in a patient with essential thrombocytosis. N Engl J Med.

[17] Morita Y, Sugiyma S, Tsuka T. Ultrasound images associated with snakebites in a Japanese Black calf. J Anim Sci Res. 2019;3(2). 10.16966/2576-6457.127.

[18] Buczinski S, Francoz D, Mulon PY (2007). Ultrasonographic diagnosis of aortoiliac thrombosis in 2 calves. J Vet Intern Med.

